# The Molecular Structures of Liquid and Glassy Nifedipine and Felodipine and Their Incorporation into PVP

**DOI:** 10.3390/ph19040638

**Published:** 2026-04-18

**Authors:** Chris J. Benmore, Stephen K. Wilke, Samrat Amin, Richard Weber, Pamela A. Smith, Stephen R. Byrn, Olivia Gibbons, Ethan Earl, Stephen Davidowski, Jeffery L. Yarger

**Affiliations:** 1 X-Ray Science Division, Advanced Photon Source, Argonne National Laboratory, Argonne, IL 60439, USA; swilke@matsdev.com (S.K.W.);; 2 School of Molecular Sciences, Arizona State University, Tempe, AZ 85287-1604, USA; samrat.amin@asu.edu (S.A.); oliviagibbons03@gmail.com (O.G.); ethance175@gmail.com (E.E.); skdavidowski@gmail.com (S.D.); jyarger@gmail.com (J.L.Y.); 3 Materials Development, Inc., Arlington Heights, IL 60004, USA; 4 Improved Pharma, West Lafayette, IN 47906, USA; pam.smith@improvedpharma.com (P.A.S.); sbyrn20@gmail.com (S.R.B.); 5 Department of Industrial and Physical Pharmacy, Purdue University, West Lafayette, IN 47906, USA

**Keywords:** nifedipine, felodipine, liquid structure, X-ray diffraction, pair distribution function, amorphous solid dispersion, computer simulation

## Abstract

**Background**: Amorphous drug formulations are commonly used to improve the solubility and bioavailability of poorly soluble molecular pharmaceuticals, yet less is known about their molecular conformations and local bonding interactions than their crystalline phases. **Methods**: High-energy X-ray diffraction structure factor measurements have been made on liquid and glassy nifedipine (NIF), felodipine (FEL), NIF 1:3 polyvinylpyrrolidone (PVP), and FEL 1:3 PVP wt.% mixtures. The corresponding X-ray pair distribution functions have been interpreted using empirical potential structure refinement using different models and density functional theory conformer calculations. **Results**: In both NIF and FEL, the NH···O inter-molecular hydrogen bonds between the pyridyl nitrogen and ester carbonyls are found to be considerably weaker than those observed in the crystalline polymorphs. For nifedipine, it is proposed that either inter-molecular NH…ON nitro bonds are present and/or a fraction (<20%) of conformational changes, with the aryl ring flipped, occur in the liquid state. For felodipine, the models indicate significant disorder associated with the methyl and ethyl side chains in the liquid state, with the main peak intensity at 3.0 Å arising from intra-molecular Cl-Cl atom pairs. When nifedipine molecules are incorporated into PVP, our models show they possess stronger NH···O bonds to the PVP polymer than felodipine molecules, which have stronger affinity for bonding to the polymer than to other felodipine molecules. **Conclusions**: The amorphous forms of both NIF and FEL show much weaker hydrogen bonding than found in their crystalline phases. Liquid NIF also exhibits configurations which are not observed in the crystal phases.

## 1. Introduction

**Figure 1:**
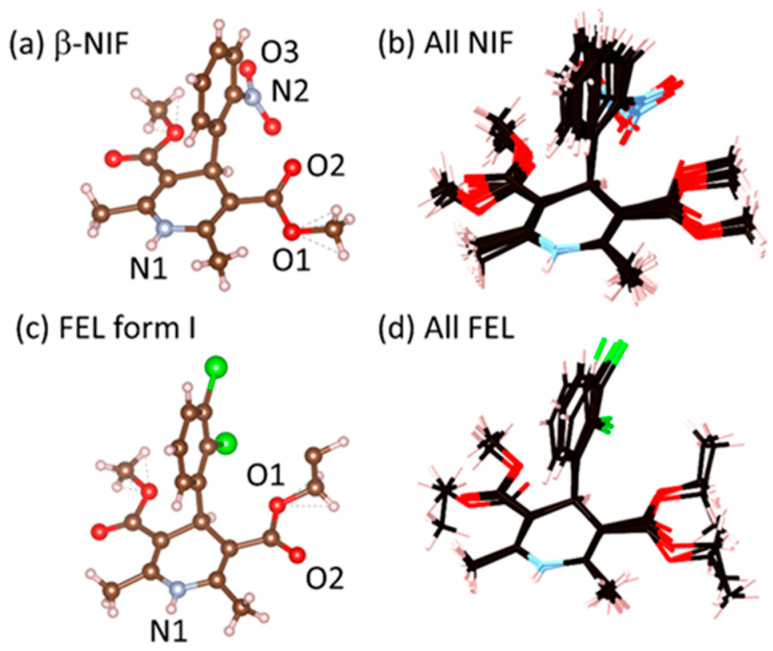
Molecular conformations associated with (**a**) β-nifedipine, (**b**) nifedipine molecules overlaid from all crystal structures, (**c**) felodipine S-enantiomer only, and (**d**) felodipine molecules overlaid from all crystal structures with both R- and S-enantiomers. Both molecules contain carboxylic acid groups (with oxygens denoted O1 and O2) and a dihydropyridine group with the nitrogen N1. Felodipine contains a dichlorophenyl group. In its place, nifedipine has a nitro group with the nitrogen denoted N2 and oxygens O3.

Nifedipine and felodipine are both calcium channel blockers that act as strong cardiac medicines, used to treat hypertension and high blood pressure by relaxing blood vessels [1,2]. Nifedipine (NIF) has six polymorphs and serves as a model for studying the crystallization of amorphous organic glasses [3], see Figure 1. The polymorph that grows preferentially from the liquid NIF depends on the temperature. The thermodynamically stable α-phase of NIF is observed to grow only above 393 K. Below 315 K, liquid NIF turns to glass, and its crystallization yields the β phase [4]. Felodipine (FEL) is a chiral drug with four confirmed polymorphs, where the activity [5], toxicity and adverse reactions of its enantiomers can vary [6], i.e., the R-enantiomer of felodipine is more likely to cause adverse reactions than the S-enantiomer. Difficulty in obtaining the pure glassy form of FEL has been reported in a recent X-ray pair distribution function (PDF) study, such that the amorphous form is commonly stabilized by mixing with the polymer polyvinylpyrrolidone (PVP) [7].

Amorphous solid dispersions (ASDs) are commonly used to improve the solubility and bioavailability of poorly water-soluble molecular pharmaceuticals over their crystalline forms, which arises from the variation in local bonding interactions. Spectroscopy measurements have shown the formation of hydrogen bonds between the amino groups of FEL and the carbonyl oxygens in PVP [8]. The presence of hydrogen bonding interactions in FEL-PVP solid dispersion systems has been attributed as the main cause of the dissolution enhancement of FEL compared to NIF in the bloodstream. The crystallization tendency of NIF and FEL as model pharmaceuticals in the supercooled state has also been investigated by Gerges et al. [9], using molecular dynamics to determine the relations between structure and their physical properties. In this study, we use high-energy X-ray diffraction experiments combined with empirical potential structure refinement (EPSR) modeling to investigate the intra- and inter-molecular structures, the degree of hydrogen bonding, and interactions of NIF and FEL molecules when they are incorporated into PVP.

## 2. Results and Discussion

### 2.1. NMR Spectroscopy

**Figure 2:**
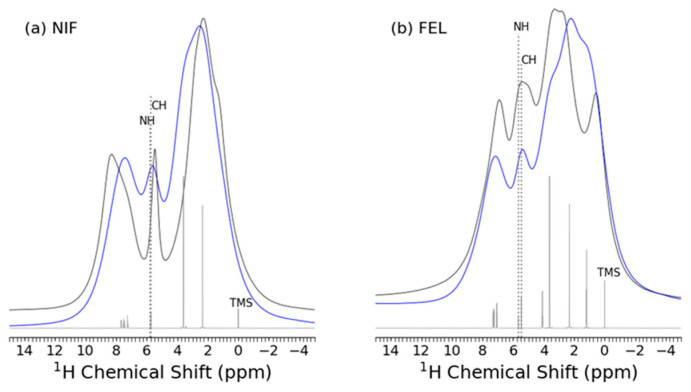
1H ssNMR spectra for (black) crystalline and (blue) amorphous (**a**) nifedipine (NIF) and (**b**) felodipine (FEL) at a MAS speed of 35 kHz, as well as the corresponding (grey dashed) 1H liquid-state NMR spectra for NIF and FEL dissolved in CDCl3, with TMS as 0 ppm reference.

The nuclear magnetic resonance (NMR) spectroscopy results for both crystalline and amorphous NIF and FEL are shown in Figure 2. A small but reproducible shift and broadening in the 1H resonance of the overlapping NH and CH region (labeled) is observed in amorphous NIF. It is also interesting to note the opposite chemical shift behavior of the aliphatic region between crystalline and amorphous NIF and FEL.

### 2.2. Nifedipine X-Ray Results

**Figure 3:**
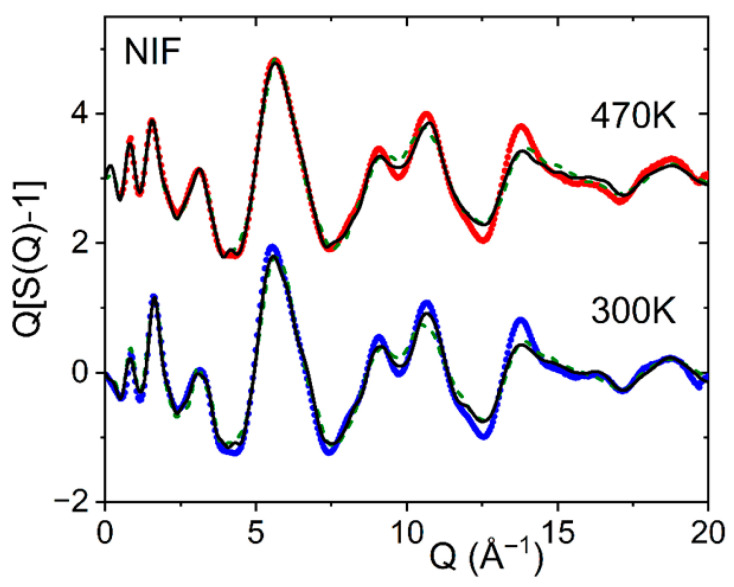
Measured X-ray structure factors Q[S(Q)-1] for glassy nifedipine at 300 K (blue circles) and liquid at 470 K (red circles, shifted by +3), compared to EPSR starting models set up using the α-conformer (green dashed line) and the β-conformer molecule (black solid line).

**Figure 4:**
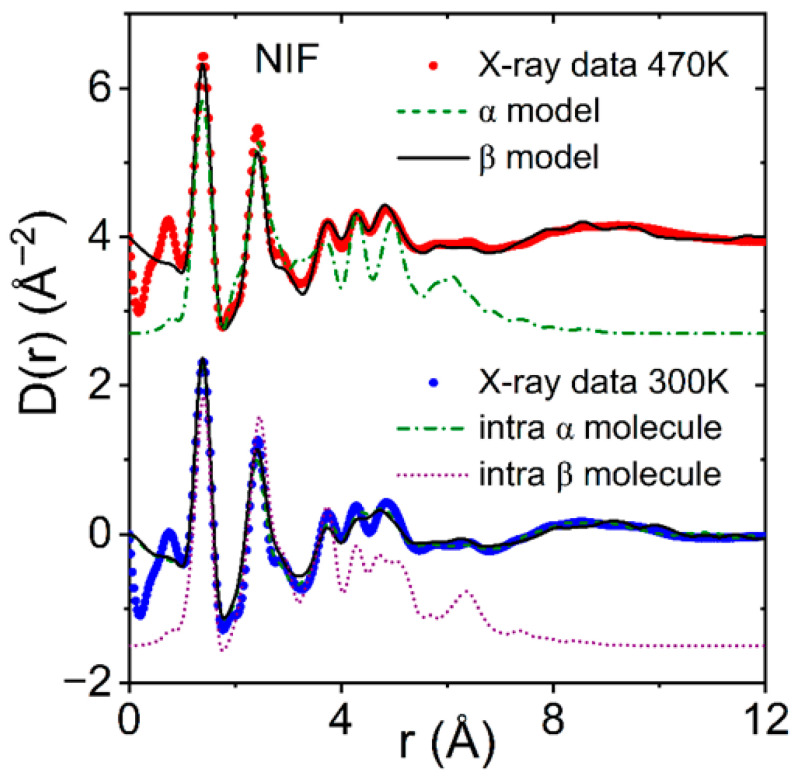
The differential X-ray pair distribution functions D(r) for glassy nifedipine at 300 K (blue circles) and liquid at 470 K (red circles, shifted by +4) compared to EPSR models using the β-conformations as the starting molecules (black solid lines). The intra-molecular contributions from a single α-conformation molecule (green dash-dot line) and single β-conformation molecule (magenta dotted line) are also compared.

The α-phase is the most stable NIF form and can be obtained using both solution crystallization and crystallization from the melt. The structure of the metastable β polymorph of nifedipine (NIF) has two molecules with similar conformations, but the molecular conformations are significantly different from α [3]. Our EPSR fits were based on the α and β molecular starting conformations, but both had five rotations enabled to allow some internal intra-molecular flexibility. The results are compared to the liquid and glassy X-ray structure factors and pair distribution functions measured at 430 K and 300 K in Figure 3 and Figure 4, respectively. Only slightly better fits to the NIF X-ray pair distribution functions for distances below 6 Å were found when the starting conformer was from the β-form compared to the α-form (determined from the goodness-of-fit R-factor where R_α_ = 0.0050 compared to R_β_ = 0.0028). This is consistent with density functional theory calculations that predict that most of the NIF crystal conformers have similar energies [10].

Moderately strong intermolecular hydrogen bonds of the type NH···O (ester carbonyl) are present in both the crystalline α and β NIF polymorphs. In α NIF, the NH···O bond connects neighboring molecules, with the N1···O2 separation being 3.028 Å and the NH···O angle being 160.3°, to form infinite hydrogen-bonded chains along the *b* axis. In β NIF, the NH···O bond connects two symmetry-independent molecules A and B to form infinite chains of the type ABAB along the [01$\bar{1}$] direction. At 296 K, the A → B and B → A hydrogen bonds are described by NH···O separations of 3.040(3) and 3.093(3) Å as determined from single crystal diffraction [3], but are reported to be 3% longer using powder diffraction [11]. High-resolution NMR studies of nifedipine in solution have also shown the presence of NH···O hydrogen bonds (supplemental information), but the screening of the hydrogen rigidly connected between the two rings prevents this atom from participating in hydrogen bonds [12].

**Figure 5:**
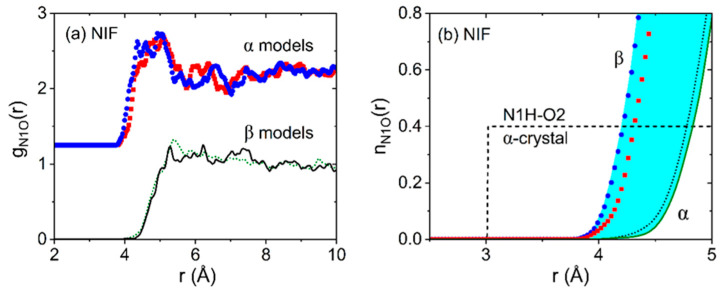
(**a**) Liquid and glassy nifedipine bonding represented by the N1-O partial pair distribution functions from our EPSR models at 300 K, where O represents the HB acceptors O2 plus O3 (α-conformation blue circles shifted by +1, β-conformation black line), and 470 K (α-conformation red squares shifted by +1, β-conformation dotted green line). (**b**) The corresponding n_NO_(r) running coordination numbers at temperatures of 300 K (α blue circles, β black dotted line) and 470 K (α red squares, β black solid line) compared to the N1-O2 coordination number found in the α-crystal at 297 K (green dashed line). The blue shading illustrates the range of hydrogen bonding found in our models compared to the form I crystal.

In our liquid and glassy EPSR NIF models, obtained using either α or β molecule starting conformations, the N1H···O2 bonds are severely weakened such that the N1···O2 distance is extended to distances ≥4 Å. In addition, we find a similar number of N1H···O3 bonds between the amino and nitro groups, as well as closely packed (non-bonded) nitro-nitro distances. At 430 K and 300 K, both models show the shortest N1H···O3 distances occur at ~3.8 Å between neighboring molecules, see Figure 5. In comparison, hydrogen bonds in the crystalline forms occur at 3.02 Å in form I crystal, 2.9 Å in form II, 3.0 Å in form III and 2.9 Å in form IV [13]. The N-O coordination numbers are higher for the β molecular starting conformation models compared to the α starting conformation models, yielding n_N1O_(r) = 0.4 at distances of r = 4.25 Å for the β model, and r = 4.8 Å for the α model. Both models indicate the liquid and glass N1H-O bonding to be similar with substantially weaker bonds than found in the most stable a-crystal form, see Figure 5b.

Our ssNMR data collected on amorphous and crystalline NIF and FEL revealed substantial overlap between the NH···O=C shift and the NH···O=N shift, which did not change between the crystalline and amorphous samples [14]. This made identification of the different hydrogen bonding interactions inconclusive. However, our EPSR results are consistent with the recent proposal that the NIF molecule can possess a diverse number of intermolecular hydrogen bonding configurations [15]. Since the nitro group can form both single-sided and bifurcated hydrogen bonds, that can offset the effects of close packing.

### 2.3. Possibility of Other Conformers

**Figure 6:**
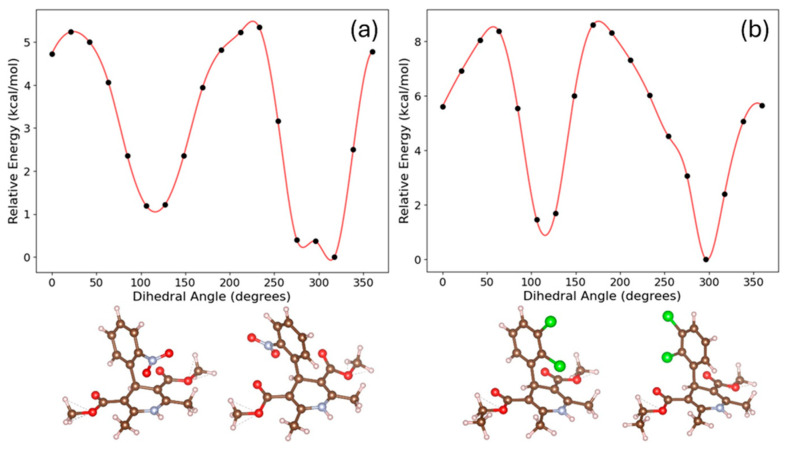
Density functional theory (wB97M-D3 functional and TZVP basis set) calculations of the relaxed dihedral angle scans of (**a**) nifedipine (NIF) and (**b**) felodipine (FEL) aryl rings using ORCA 6.1.1 [16,17,18].

Alternate structural explanations should also be considered in the high-temperature melt. Density functional theory conformer searches for single molecules of NIF and FEL performed in Rowan and ORCA indicate additional conformations under 3 kcal/mol [14,16,17,18]. Although both molecules have their lowest energy state with the primary substituted ring (NIF—NO_2_ substituted and FEL—2Cl substituted) moieties being opposite of the NH, both have conformations where the NO_2_ moiety in NIF and Cl moiety in FEL are flipped ~180° and are close in proximity to the NH (see Figure 6). This aryl ring-flip with two stable conformations could potentially have significant impact on the high-temperature molecular liquid state structure and could contribute to the molecular structure associated with the X-ray pair distribution EPSR model interpretation. Hence, instead of being inter-molecular N1H…ON interactions from the lowest energy conformation, there could be some intramolecular interactions of the second conformation, which would be much more prevalent at high temperature and potentially frozen into the glass structure below T_g_.

Although there is no direct experimental evidence for the existence of a second conformer in liquid NIF, we have tested the possibility by fitting various populations of the two conformers to the X-ray structure factor using EPSR. The results indicate that up to ~20% of the second conformer (with the aryl ring flipped) may occur in liquid NIF at 470 K. This was mainly determined by the fit to the magnitude and shape of the first sharp diffraction peak, and the scaling factor used to adjust the normalization of the X-ray data in EPSR model simulations [14].

### 2.4. Felodipine X-Ray Results

**Figure 7:**
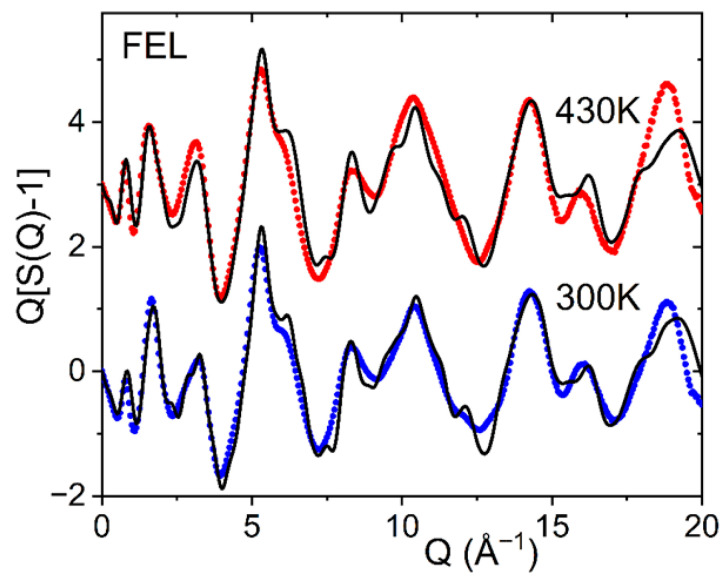
Measured X-ray structure factors Q[S(Q)-1] for glassy felodipine at 300 K (blue circles) and liquid at 430 K (red circles, shifted by +3) compared to EPSR models obtained using a racemic mixture of R- and S-enantiomers (black solid line).

Crystallization and polymorphism of felodipine have been investigated using single-crystal X-ray diffraction by Surov et al. [13]. Form I is the most thermodynamically stable phase and racemic, with two enantiomers, S-felodipine and R-felodipine. Form II shows the highest solubility and intrinsic dissolution rate, consistent with the lowest thermodynamic stability. Forms I, II, and III are all monotropically related. In all polymorphs, the molecular conformation is comparable, with the dichlorophenyl group perpendicular to the plane of the remainder of the molecule. The orientation of the methyl and ethyl ester groups is such that the carbonyl O atom points toward the methyl groups on either side of the pyridyl N1 atom in forms I and II, while the ethyl ester adopts the opposite orientation in forms III and IV. The pyridyl ring in form III is distorted and shows some disorder in the methyl and ethyl ester groups. Figure 7 shows EPSR fits to the liquid and glassy X-ray structure factors and pair distribution functions measured at 470 K and 300 K based on a racemic mixture of S- and R-enantiomers. The disorder associated with the methyl and ester groups is reflected in the magnitude and shape of the second peak in D(r) encompassing the 2.5–3.1 Å region, compared to enantiomer configurations found in forms I and II.

**Figure 8:**
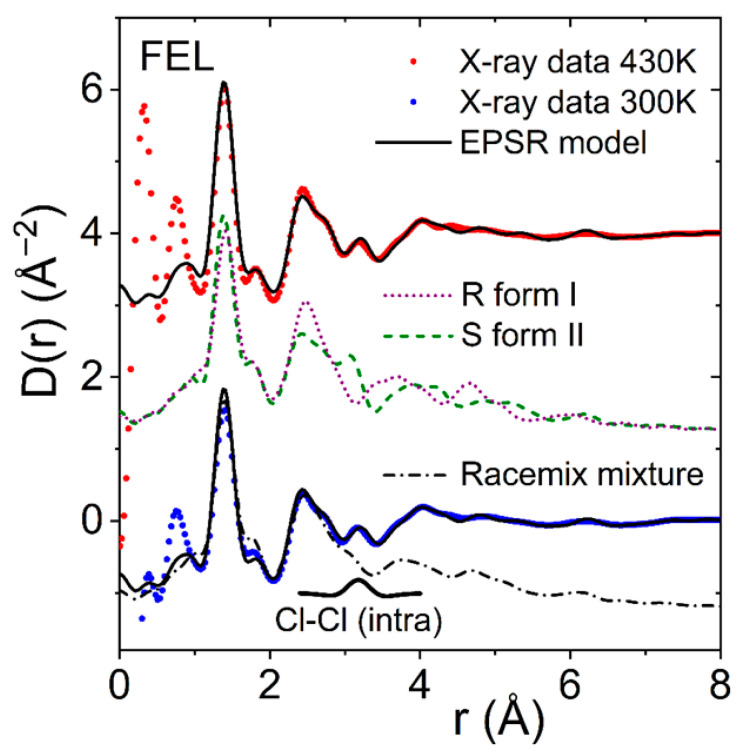
The differential X-ray pair distribution functions D(r) for glassy felodipine at 300 K (blue circles) and liquid at 430 K (red circles, shifted by +4) compared to EPSR models using a racemic mixture of R-and S-enantiomers (black solid lines). Here, interchange of the methyl and ethyl chains inverts the chirality of the felodipine molecule. Disorder associated with variations in the orientations of these chains is demonstrated through a comparison of the intra-molecular contributions from a single R-enantiomer molecule from form I (magenta dotted line) and S-enantiomer molecule from form II (green dashed line) shifted by +1. Also shown is the X-ray weighted intra-molecular Cl-Cl partial pair distribution function in the region of 3.1 Å (black solid line, see text).

Geddes et al. [7] described amorphous FEL as “unobtainable”, so extrapolated from FEL-PVP mixtures to obtain the X-ray PDF of pure amorphous FEL. The main difference in preparation was that these samples were extruded and manually milled into a fine powder, whereas our samples were melt-quenched and measured as-is in the capillary. Differences between amorphous FEL and the crystal in the ~3.0 Å region were attributed to the inaccessibility of conformational rotamers [7]. Supporting ab initio molecular dynamics simulations suggest a close structural parallel between felodipine conformations sampled in its melt and amorphous states, and indicate the presence of ethoxy rotamers that are forbidden in the crystalline form, appearing as Cl-ethoxy pair correlations in the X-ray PDF. Our EPSR models show that while Cl-ethoxy rotamer correlations exist in this region, and do vary due to side-chain disorder in the liquid state, they are much more diffuse compared to the relatively narrow Cl-Cl peak at 3.0 Å in the PDF, that corresponds primarily to the relatively rigid intra-molecular atom Cl-Cl distance. Indeed, the Cl-Cl distance is found to be slightly further apart (0.05 Å) in the liquid state compared to the crystal, corresponding to a slightly larger ÐCCCl angle of 4º within the dichlorophenyl ring (see Figure 8).

**Figure 9:**
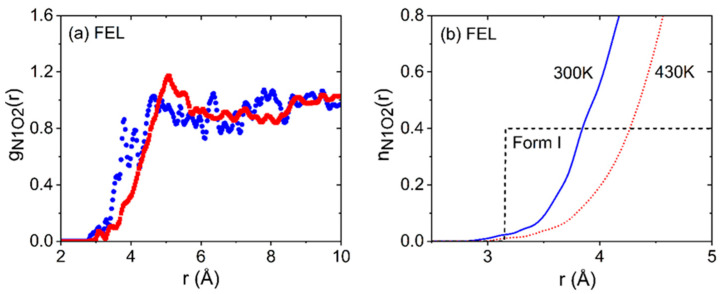
(**a**) Glassy and liquid felodipine hydrogen bonding N1H···O2 partial pair distribution functions from our EPSR models of racemic mixtures of R- and S-enantiomers at 300 K (blue circles) and 470 K (red squares), respectively. (**b**) The corresponding n_N1O2_(r) running coordination numbers at temperatures of 300 K (solid blue line) and 470 K (dotted red line), compared to that of the form I crystal at 123 K (black dashed line).

All four FEL crystal structures contain NH···O hydrogen bonds from the pyridyl N atom to the carboxyl O atoms of the methyl/ethyl ester groups [13], with intermolecular NH···O bonds in the range 2.9–3.2 Å. In forms III and IV, these define distinct linear ribbons. In III, all molecules in the ribbons are along the b-axis. In IV, adjacent molecules are turned so that their pyridyl planes lie approximately perpendicular to each other. The ribbons in form III comprise exclusively molecules of one enantiomer, while those in form IV contain molecules of opposite chirality alternating along a given chain. In forms I and II, the hydrogen bonds define “zig-zag” arrangements, with form I containing bifurcated NH···O interactions. In forms III and IV, the pairs are linked into linear ribbons by the NH···O hydrogen bonds, and the polymorphism corresponds to turning every second dimer in each ribbon by ca. 90° in form IV, compared to its orientation in form III. Our racemic liquid EPSR liquid and glassy FEL models are similar to the results for NIF in that the NH···O intermolecular hydrogen bonds between FEL molecules are found to be largely broken or at least substantially weakened compared to the crystals. The g_NO_(r) partial pair distribution functions are shown in Figure 9 and indicate some temperature dependence between the liquid and glassy states, with slightly stronger interactions at 300 K compared to 430 K. More importantly, the corresponding N-O running coordination number for FEL reaches a value of n_NO_(r) = 0.4 at r = 3.8 Å for the glass, decreasing to 4.3 Å in the liquid, whereas this occurs at a considerably shorter distance of r = 3.16 Å in crystalline form I, indicating a substantial number of weak or non-hydrogen bonded NH- interactions present in liquid and glassy FEL.

**Figure 10:**
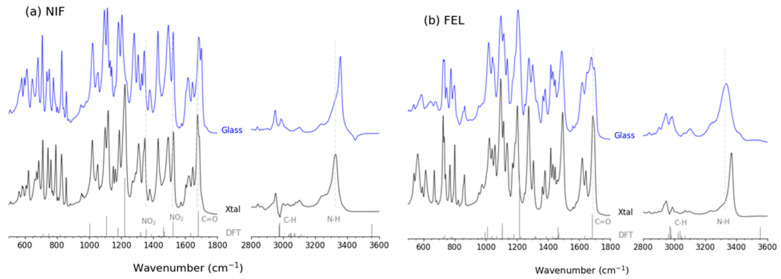
ATR FT-IR spectra of crystalline (black) and amorphous (blue) (**a**) nifedipine and (**b**) felodipine. The lines (gray) are the calculated scaled (0.975) frequencies from molecular optimized DFT calculations. The 2800–3600 cm^−1^ region has a ×2 zoom.

Previous Raman and infra-red (IR) spectroscopy measurements have reported that the average strength of the NH stretch associated with intermolecular hydrogen bonds in the amorphous forms of NIF and FEL is similar. However, it has been argued that the average strength of hydrogen bonding in the amorphous form of FEL is stronger than in its crystalline form, due to the peak shift to higher wavenumbers [19,20]. Our measurements on crystalline and amorphous NIF and FEL have reproduced these FT-IR spectra first reported by Marsac et al. [19], see Figure 10. Although the strength of intermolecular hydrogen bonding interactions is often characterized by a peak shift in IR spectroscopy, in amorphous systems, this can be ambiguous because there are a distribution of configurations from broad overlapping peaks, making specific identification problematic. Indeed, this interpretation is inconsistent with the results of our X-ray-derived EPSR models presented above. The N1-O pair distribution functions and associated coordination numbers found in our EPSR models indicate that the FEL crystal has, on average, stronger hydrogen bonds, and considerably more of them, than the glassy form. To test if this was due to the minimum distance constraints imposed in glassy FEL (2.9 Å for N-O contacts), they were removed, and the simulation re-run. Although a slightly higher N-O coordination at shorter distances was observed, no stronger bonds were observed in glassy FEL. Similarly, no changes were observed when the minimum distance constraints imposed in glassy NIF were removed.

Consequently, we investigated other possible explanations. Previously, the small peak around 3420 cm^−1^ has been assigned to a small fraction of “free” (non-hydrogen bonded) N-H stretching groups, and the principal peak at 3345 cm^−1^ associated with intermolecular NH···O hydrogen bonds [19,21]. Given the predominance of non- or weakly hydrogen-bonded FEL molecules in our glassy FEL models, we suggest the free N-H stretch is the main contribution to the principal peak in the FT-IR spectra since it lies well within the N-H stretching region [21]. In addition, our EPSR models are consistent with the physical properties, since the amorphous form of FEL is known to be 6–8% lower in density than the crystalline form, consistent with stronger inter-molecular bonds and closer molecular packing in the crystals.

### 2.5. The First Sharp Diffraction Peak

**Figure 11:**
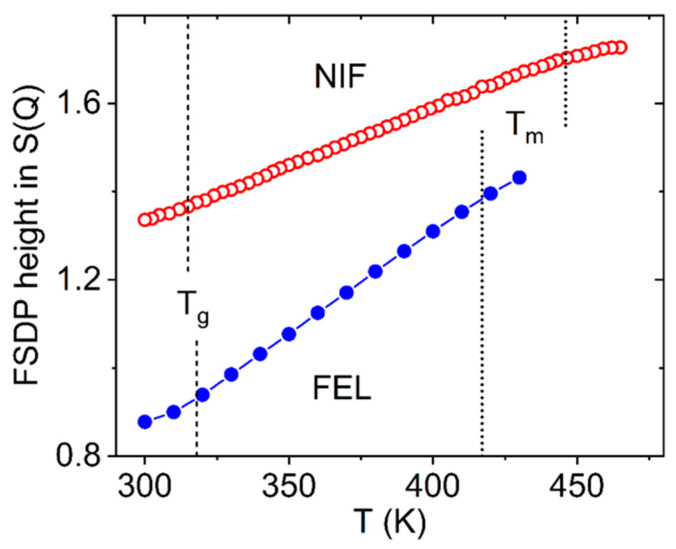
The first sharp diffraction peak intensities as a function of temperature for nifedipine (open red circles) and felodipine (closed blue circles). The melting temperatures T_m_ (dashed lines) and glass transition temperatures T_g_ (dotted lines) are also shown.

A first sharp diffraction peak (FSDP) is commonly associated with intermediate range order in a liquid. An FSDP is known to exhibit anomalous behavior with temperature, in an opposite manner to other peaks in the S(Q). Specifically, the intensity of the FSDP increases with increasing temperature, whereas other peaks naturally broaden at higher temperatures, as expected from growing Debye–Waller factors, see Figure 11. Price et al. [22] have shown that the FSDP originates from the competition between the rapidly decreasing form factor from the intra-molecular structure factor and a rising inter-molecular structure factor associated with the packing of the molecules. As the temperature of the molecular liquid is increased, the lower density of the fluid generally leads to a shift in the inter-molecular structure factor corresponding to the packing of molecules. Structural changes at higher temperature due to reduced steric hindrance and/or competition between optimal intra-molecular conformation and inter-molecular bonding may also occur.

### 2.6. NIF/FEL Mixtures with PVP

**Figure 12:**
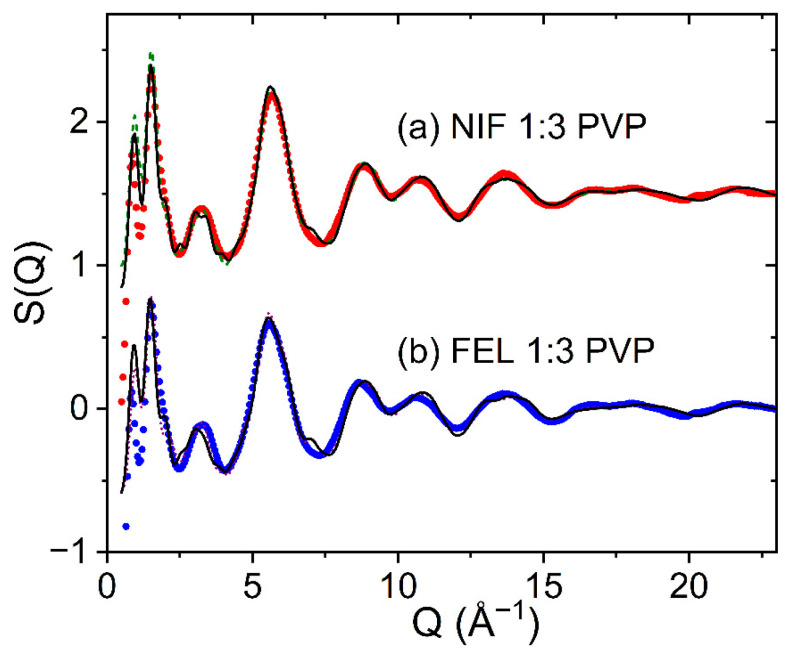
The measured X-ray structure factors for (a) amorphous NIF 1:3 PVP (red circles) compared to EPSR models comprising α-conformation molecules (green dashed line) or β-conformation molecules (black solid line), shifted by +1.5; (b) amorphous FEL 1:3 PVP (blue circles) compared to an R-enantiomer (dotted magenta line) and S-enantiomer model (solid black line), shifted by −1.

Hydrogen bonding patterns of amorphous solid dispersions of FEL and NIF with various binders have been studied via FTIR and ssNMR analyses, to shed light on how the structure of the glassy forms can stabilize against crystallization [3,23]. The influence of a polymer on the crystallization rate of amorphous drugs is typically described in terms of properties of the amorphous metastable form, such as the glass transition temperature T_g_, the molecular mobility, and the bonding interactions occurring between the drug and the polymer [19]. Interestingly, Marsac et al. [19] have shown that NIF crystallizes more readily than FEL in PVP despite having a similar T_g_ and molecular mobilities. NMR relaxometry studies on FEL-PVP amorphous solid dispersions have found that although PVP cannot prevent phase separation of felodipine under high humidity, the presence of PVP can minimize the crystallization of amorphous FEL domains [24]. For our X-ray analysis, Figure 12 shows the EPSR fits for NIF 1:3 PVP and FEL 1:3 PVP models, using the α or β NIF conformations and the R- or S- FEL enantiomers, respectively. Both sets of EPSR models give reasonable fits to the X-ray diffraction patterns.

**Figure 13:**
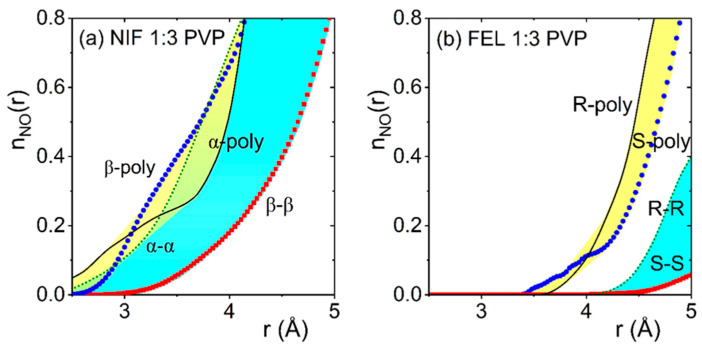
The n_NO_(r) running coordination number for the intermolecular N-O bonds in (**a**) NIF 1:3 PVP at 300 K, where O either corresponds to an oxygen on the NIF molecule (α-conformation red squares, β-conformation green dashed line) or an oxygen on a PVP monomer (α-conformation blue circles, β-conformation black solid line); (**b**) FEL 1:3 PVP at 300 K, where the O corresponds to an oxygen on the ester carbonyl (R-enantiomer green dashed line, S-enantiomer red squares) or an oxygen on a PVP monomer (R-enantiomer black solid line, S-enantiomer blue circles). The yellow shading illustrates the drug–polymer regions, the blue shading the drug–drug region, and green shading is the overlap.

**Figure 14:**
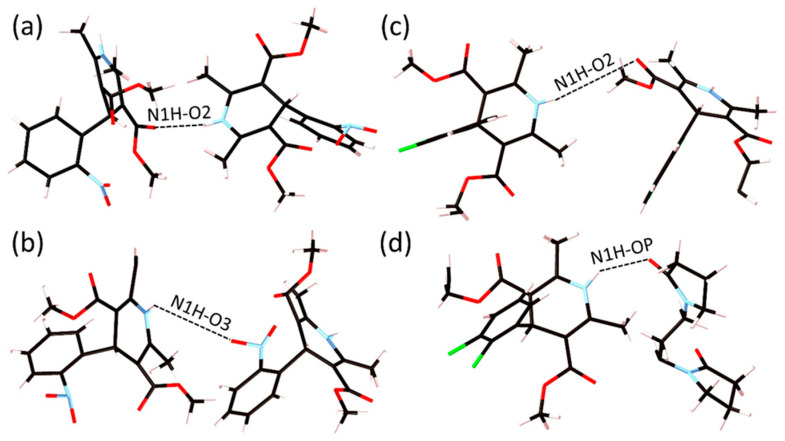
Inter-molecular hydrogen bonding interactions found in our EPSR models between the amino group on an (**a**) NIF molecule and an adjacent NIF carbonyl oxygen, (**b**) NIF molecule and a nitro oxygen on another NIF molecule, (**c**) FEL molecule and a carbonyl oxygen on another FEL molecule, (**d**) FEL molecule and a carbonyl oxygen on a section of PVP.

Our EPSR hydrogen bonding N-O running coordination numbers for amorphous NIF 1:3 PVP in Figure 13 show that both the α- and β-conformation NIF molecules have a preference for bonding to the PVP polymer or α–α molecule bonding interactions at shorter distances of 2.6–3.0 Å, over β–β hydrogen bonds at ~3.6 Å. The results are more distinct in the FEL N-O running coordination numbers for S- and R-enantiomers, which exhibit hydrogen bonding interactions at a peak distance of 4.7 Å with the PVP polymer than between like FEL molecules. Crystallization of NIF in PVP glasses has been found to yield only the β polymorph, in preference to other polymorphs [3,25,26], despite the a-polymorph being the most stable. A graphical summary of the intermolecular hydrogen bonding interactions found in our EPSR models is shown in Figure 14. Despite the variations between EPSR models, there is a strong preference for FEL molecules to bond to PVP (yellow regions) rather than themselves (blue regions), whereas for NIF, the distinction is less pronounced, which could potentially lead to nano-segregation. Ultimately, preferential bonding between NIF drug molecules could act as a nucleation seed, promoting crystallization by encouraging ordered arrangements between drug molecules. This ordering can increase the nucleation rate, rather than forcing the mixture to remain amorphous.

## 3. Materials and Methods

The nifedipine (Millipore-Sigma ≥98% purity, Milwaukee, WI, USA) and felodipine (U.S. Pharmacopeia ≥99.5%, Rockville, MD, USA) were purchase and determined to be in a standard crystalline form with high purity. It was this material that was directly heated to the liquid state and quenched to a glass. The synthesis of NIF and FEL ASDs was performed using 10 kDa polyvinylpyrrolidone (PVP, Sigma-Aldrich, Milwaukee, WI, USA) and high-purity acetone.

### 3.1. Preparation of Drug-Polymer Amorphous Solid Dispersions

Drug–polymer mixtures of NIF 1:3 PVP and FEL 1:3 PVP in wt.% were dissolved together in HPLC-grade acetone, roto-vapped, and vacuum-dried to make an amorphous solid dispersion. The PVP used was Kollidon 17 PF (MW ~9000 BASF, Florham Park, NJ, USA), which was vacuum-dried prior to use. The resulting powder was pressed into a pill shape using a hydraulic press to 5 kbar. Since stored samples containing PVP can pick up additional water from the atmosphere, the samples were dried in situ for 1 h on the beamline prior to the x-ray measurements.

### 3.2. Thermal Analysis

Differential scanning calorimetry (DSC) measurements were performed on a TA Instruments Discovery 2500 DSC (New Castle, DE, USA) with a constant dry nitrogen flow >200 mL/min. The baseline, forward, and reversing heat capacity were calibrated using sapphire disc standards, while the cell constant was calibrated using In wire standard (Strem chemicals, 99.9985%, Newburyport, MA, USA). DSC thermograms (supplemental information [14]) of nifedipine and felodipine were measured in sealed aluminum hermetic pans with a heating rate of 10 K/min. The results confirmed that nifedipine melts at T_m_ = 446 K and has a glass transition temperature of T_g_ = 315 K, and felodipine exhibits these transitions at T_m_ = 415 K and T_g_ = 316 K, in good agreement with the literature [27].

### 3.3. Vibrational Spectroscopy

Attenuated total reflection (ATR) Fourier transform infrared spectroscopy (FT-IR) was performed using a Perkin Elmer spectrum two spectrometer, (Shelton, CT, USA). A standard polystyrene film was used for calibration, and isopropanol was used for cleaning the diamond ATR. IR spectra were collected in ambient conditions (298 K, 1 atm, in air) from 400 to 4000 cm^−1^ in increments of 4 cm^−1^ for a total of 64 scans, with a zero-baseline correction applied at lower frequencies by interpolation.

### 3.4. Nuclear Magnetic Resonance (NMR) Spectroscopy

Solution-state NMR data were collected on a Bruker 500 MHz NEO spectrometer (Billerica, MA, USA) with a 5 mm iProbe with NIF, FEL and ASDs dissolved in millimolar ratios into d_6_-dimethyl sulfoxide (DMSO) with tetramethylsilane (TMS) as the chemical shift reference. The solid-state NMR data were collected on a Varian VNMRS (9.4 T) operating at a Larmor frequency of 400 MHz and 100 MHz for ^1^H and ^13^C, respectively. A 1.6 mm Varian T3 high-speed magic angle spinning (MAS) probe was employed with a common MAS speed of 35 kHz. Liquid-state NMR data were collected on a Bruker 500 MHz NEO spectrometer with a 5 mm iProbe, and solid-state NMR data were collected on a 400 MHz Varian VNMRS system with a 1.6 mm triple resonance HXY high-speed magic angle spinning (MAS) probe. The direct detection 1H ssNMR data were collected with a recycle delay of 120 sec for crystalline samples and 10 s for amorphous samples, 8 scans, and a MAS rate of 35 kHz. The samples were spun using cooled bearing gas to maintain a temperature below the T_g_ of the amorphous samples. The sample temperature was calibrated using lead nitrate, and the sample temperature was determined to be 282 K. The instrument was set to regulate at 268 K. The 1H-13C cross polarization spectra for NIF and FEL and the NIF 1:3 PVP and FEL 1:3 PVP amorphous solid dispersions are shown in the supplementary information [14].

### 3.5. High-Energy X-Ray Diffraction (HE-XRD)

High-energy X-ray diffraction experiments were performed on NIF and FEL at the Advanced Photon Source on beamline 6-ID-D, (Argonne National Laboratory, Lemont, IL, USA) using an incident X-ray beam of wavelength 0.1534 Å (80 keV). The beam of 0.5 mm × 0.5 mm in size impinged on powdered samples contained in thin-walled (0.1 mm) capillaries of 2.0 mm diameter. The scattered intensity was measured with a Varex CT4343 area detector (Salt Lake City, UT, USA) placed ∼344 mm downstream of the sample and calibrated using a CeO_2_ powder standard (NIST) to provide a wide Q range of 0.6–25.0 Å^−1^. Liquid nifedipine was measured using a thermoelectric stage at 470 K and supercooled to the glassy state at 300 K. Similarly, liquid felodipine was measured at 430 K, and the glass at 300 K. Both were measured for five 60 s scans, during which time no degradation of the samples was observed. The X-ray experiments conducted on the 1:3 NIF:PVP and FEL:PVP were performed in a Linkam stage at 10% RH after drying for 1 h. No evidence of the presence of water was found. The X-ray data were analyzed using the Fit2D software (version 18-beta) [28] and corrected for flat field, polarization, detector rotation, and tilt. The program PDFgetX2 (version 1.0) [29] was used to correct for background, oblique incidence, absorption, and detector efficiency effects and normalize the X-ray intensity data to the sample self-scattering in absolute electron units. The X-ray total structure factors S(Q) and the corresponding differential pair-distribution function [$D(r)\;=\;4\pi r^{2}\left[G\left(r\right)-1\right]$ have been extracted using standard methods using the Hannon–Howells–Soper formalism [30].

### 3.6. Computational Modeling

Density functional theory (DFT) calculations were performed at the ground state (in vacuo) using the Gaussian 16 software package [31] with the B3LYP functional and 6-311++G(2d,3p) basis set [32]. The optimized molecular structures were subsequently analyzed by GaussView 6 to derive the geometric parameters, electronic properties, energetic characteristics, and IR and Raman vibrational modes.

Empirical potential structural refinement (EPSR) is a reverse Monte Carlo method used to find three-dimensional arrangements of molecules that fit the measured X-ray diffraction data [33,34]. Starting with reference Lennard Jones potentials and partial charges to imitate bonding interactions, the inter-atomic potentials are iteratively altered until agreement is reached between the models and the X-ray S(Q) pattern. Details of the EPSR modeling process applied to liquid and glassy pharmaceutical and PVP molecules based on high-energy X-ray diffraction have been described previously [35,36,37].

NIF is a flexible molecule with four major torsional angles [10]. Torsions occur between the dihydropyridine ring and the nitro group, and the dihydropyridine ring and the ester groups, see Figure 1. For the ester torsions, the cis/trans conformer produces the lowest-energy crystals, which occur in five of the polymorphs, followed by the cis/cis conformer, which occurs in one polymorph [6]. The N1H group is the only hydrogen bond donor, and the ester O2 and nitro O3 groups are hydrogen bond acceptors. Our EPSR simulations of nifedipine were performed on 64 molecules within a cubic box using the most stable α-form conformation at 297 K (BICCIZ07 from the Cambridge Crystallographic database [38]) and the β-form (BICCIZ03). Partial charges were assigned based on the molecular dynamic simulations by Gupta & Kothekar [39], and standard optimized potentials for liquid simulations as shown in Table 1 [40]. Based on the different crystalline forms (α, β, γ, δ and the disordered counterparts β’ and γ’), a semi-rigid model with five rotations was enabled, associated with the methyl and nitro groups. The simulation was initiated by a random arrangement of molecules at low density, and the box size was gradually decreased until agreement with the first sharp diffraction peak height was obtained, because it was very sensitive to density (see discussion in next section).

Final dimensions of the cubic box of length 30 Å corresponded to number densities in the range 0.105–0.110 atoms Å^−3^ (1.40–1.47 g × cm^−3^) at 300 K. This is slightly higher than the density of amorphous NIF reported by Saraf et al., of 1.34 g × cm^−3^ [41] and of the crystalline forms, of ~1.38 g × cm^−3^ at 296 K [10], and may be an artifact of the small number of molecules in the simulation box or due to a small conformational variation in the molecular shape that is not accounted for in our models. The higher density does not have a significant effect on local coordination numbers or distances in our EPSR models; rather, it affects the packing of the molecules in the simulation box, which cannot be completely filled with molecules at the specified experimental density. A discrepancy in the density between the EPSR model and experiment shows up in the S(Q) fit at low-Q values, typically in the region Q < 2 Å^−1^. If the density of the model is too low, there is a sharp rise at low-Q. This is because the EPSR simulation preferentially tries to fit the molecular conformation and local packing requirements dominated by the majority of the S(Q) pattern, which dominates at Q > 5 Å^−1^.

Similarly, the EPSR simulations of felodipine were performed on 64 molecules within a cubic box of length ~30 Å, using a number density of 0.093 atoms Å^−3^ (1.35 g × cm^−3^) at 300 K. This value is close to the reported density of 1.33 g × cm^−3^ for amorphous FEL [42], and significantly lower than the crystalline densities which lie in the range 1.42–1.45 g × cm^−3^ [13], The same partial charges and Lennard-Jones starting parameters were used as for nifedipine, except for the dichlorophenyl group (see Table 1) [40]. Based on the different crystalline felodipine forms I, II, III, and IV [13] (possibly V [43]), a semi-rigid model was constructed with the only rotations allowed being associated with the two ester groups, the nitro group, and the methyl groups. Both forms I and II are racemic mixtures of two enantiomers; R-felodipine and S-felodipine (DONTIJ and DOMTIJ01 from the Cambridge Crystallographic database). Inversion of the molecule chirality is associated with the interchange of the methyl and ethyl chains. Although there are only slightly different conformations between polymorphs, associated with the orientations of these chains [13], this variation was accounted for in EPSR by mixing the form I R-enantiomer with the form II S- enantiomer. It should be noted that although the populations of molecules with R- and S- conformations do not change during the simulation, the side chains can rotate, and the angles within each molecule can vary by a few degrees. In addition, to explore the sensitivity of the EPSR method to this disorder, three different liquid models were constructed: (i) a racemic mixture of 32 R-enantiomers and 32 S-enantiomers, (ii) all R-enantiomers (form I); and (iii) all S-enantiomers (form II). Both NIF and FEL EPSR models included a minimum NH···O distance constraint of 2.9 Å based on the known crystal structures [3,13] (although these were removed in some later models, see FEL discussion section). Following the initial Monte Carlo equilibrations, the empirical potential term was automatically adjusted to improve agreement with the diffraction data. Once the goodness-of-fit parameter was minimized, structural data were collected over ensembles of at least 50,000 configurations.

EPSR models of felodipine 1:3 PVP and nifedipine 1:3 PVP mixtures were also constructed, containing nine FEL or 10 NIF molecules and 19 or 21 PVP K15 molecules, respectively (comprising five monomers [37]), in a cubic box under periodic boundary conditions. An atomic number density of 0.115 atoms Å^−3^ was used, corresponding to a cubic box size of ∼28 Å, with the simulations following procedures similar to that described for PVP-water [36] and PVP-ketoprofen mixtures [37]. For this exercise, the minority of contaminant water molecules typically present in PVP was omitted, since the focus was to compare drug–drug and drug–polymer interactions.

## 4. Conclusions and Future Work

In the crystalline forms of nifedipine and felodipine, inter-molecular hydrogen bonding patterns only occur between the pyridyl nitrogen and ester carbonyls on adjacent molecules. Based on high-energy X-ray diffraction data, we have constructed several model structures assuming a distribution of conformations similar to that found in the crystals to investigate how these interactions vary in the liquid and glassy states. Our models for both NIF and FEL show that hydrogen bonding is greatly reduced and weakened in the liquid and glassy states. In these disordered forms of nifedipine, our models also predict the occurrence of weak amino-nitro inter-molecular hydrogen bonds, and closely packed (non-bonded) nitro-nitro distances. Alternatively, we have explored the possibility of the presence of a second conformer present in liquid NIF (with the aryl ring flipped), predicted by quantum mechanical calculations. EPSR fits to the NIF X-ray structure factor data at 470 K are consistent with up to ~20% of the second conformer present.

For felodipine, the measured X-ray pair distribution functions indicate significant disorder associated with the methyl and ethyl side chains. Given the decrease in the number of NH···O inter-molecular interactions between FEL molecules upon melting the crystal, this leads to a substantial number of broken or weakly hydrogen-bonded molecules in the liquid and glass. This structural interpretation is consistent with the densities of the liquid and glassy FEL forms being lower than that of the crystal, leading to a revision of the previous assignment of peaks in the vibrational spectra. Upon mixing with PVP, our EPSR models show that both nifedipine and felodipine display an affinity for bonding to the carbonyl oxygens in PVP.

Overall, our study shows both the power and limitations of combining HEXRD with EPSR molecular modeling: namely, the ability to test and predict specific atom–atom interactions within the liquid or amorphous solid dispersion, but the inability to directly reproduce vibrational or NMR spectra. With the advent of machine learning interatomic potentials, it is anticipated that DFT conformer calculations will have a significant impact in helping overcome these limitations. Moreover, the discovery of a new conformer or inter-molecular interaction can alter the development of a drug by enabling enhanced, structure-based design to increase potency, selectivity, safety, and stability. Such a finding would allow formulation scientists to optimize drug–target binding by identifying new bioactive conformations, which can drastically improve efficacy, reduce toxicity, and aid in designing compounds that bind more tightly, typically through new hydrogen bonds or hydrophobic contacts. This could lead to hydrogen bonding optimization or even reformulation.

## Figures and Tables

**Table 1:** Partial charges and Lennard-Jones starting potential parameters for our EPSR simulations. Atoms not listed kept their default Lenard-Jones parameters in EPSR and were assigned zero charge.

Dihydropyridine	q (e)	s (Å)	e (kJ/mol)	Methyl Ester	q (e)	s (Å)	e (kJ/mol)
N (NHR)	−0.70	3.25	0.170	O (R_2_CO)	−0.47	2.96	0.210
N (Pyridine)	−0.70	3.25	0.170	C (R_2_CO)	+0.47	3.75	0.105
H (NHR)	0.000	0.00	0.000	O (ROR)	−0.40	2.90	0.140
C (Pyridine, NIF)	+0.47	3.75	0.105	C (CH_3_OR)	+0.20	3.50	0.066
C (Pyridine, FEL)	+0.13	3.55	0.700	**Nitro**			
C (CH_3_ or CH_2_)	+0.20	3.50	0.066	N (CNO_2_)	+0.45	3.25	0.170
**Dichlorophyenyl (FEL)**				O (CNO_2_)	−0.36	2.96	0.210
Cl	0.00	3.2	0.800	C (CNO_2_)	+0.18	3.75	0.105

## Data Availability

The original data presented in the study are openly available on Zenodo at https://doi.org/10.5281/zenodo.19079901.

## Supplementary Materials

The following supporting information can be downloaded at Zenodo at https://doi.org/10.5281/zenodo.19079901 (accessed on 1 April 2026). It contains (I) the 2D molecular structures of NIF, FEL and PVP, (II) differential scanning calorimetry curves for NIF, FEL and the PVP mixtures, (III) powder X-ray diffraction crystalline and amorphous patterns of NIF and FEL, (IV) nuclear magnetic resonance curves for NIF, FEL and the PVP mixtures, (V) our ab initio computation (DFT) results, (VI) EPSR fits to X-ray NIF S(Q) assuming the presence of a second conformer, and (VII) Intra-molecular NIF X-ray S(Q)s.
